# MMR vaccination induces trained immunity via functional and metabolic reprogramming of γδ T cells

**DOI:** 10.1172/JCI170848

**Published:** 2024-01-30

**Authors:** Rutger J. Röring, Priya A. Debisarun, Javier Botey-Bataller, Tsz Kin Suen, Özlem Bulut, Gizem Kilic, Valerie A.C.M. Koeken, Andrei Sarlea, Harsh Bahrar, Helga Dijkstra, Heidi Lemmers, Katharina L. Gössling, Nadine Rüchel, Philipp N. Ostermann, Lisa Müller, Heiner Schaal, Ortwin Adams, Arndt Borkhardt, Yavuz Ariyurek, Emile J. de Meijer, Susan L. Kloet, Jaap ten Oever, Katarzyna Placek, Yang Li, Mihai G. Netea

**Affiliations:** 1 Department of Internal Medicine and Radboud Center for Infectious Diseases and; 2 Radboud Institute for Molecular Life Sciences, Radboud University Medical Center, Nijmegen, Netherlands.; 3 Department of Computational Biology for Individualised Medicine, Centre for Individualised Infection Medicine (CiiM) and; 4 TWINCORE, a joint venture between the Helmholtz-Centre for Infection Research (HZI) and Hannover Medical School (MHH), Hannover, Germany.; 5 Department of Immunology and Metabolism, Life and Medical Sciences (LIMES) Institute, University of Bonn, Bonn, Germany.; 6 Department for Pediatric Oncology, Hematology and Clinical Immunology and; 7 Institute of Virology, University Hospital Duesseldorf, Medical Faculty, Heinrich Heine University Duesseldorf, Dusseldorf, Germany.; 8 Leiden Genome Technology Center, Department of Human Genetics, Leiden University Medical Center, Leiden, Netherlands.

**Keywords:** Immunology, Cellular immune response, Innate immunity

## Abstract

The measles, mumps, and rubella (MMR) vaccine protects against all-cause mortality in children, but the immunological mechanisms mediating these effects are poorly known. We systematically investigated whether MMR can induce long-term functional changes in innate immune cells, a process termed trained immunity, that could at least partially mediate this heterologous protection. In a randomized, placebo-controlled trial, 39 healthy adults received either the MMR vaccine or a placebo. Using single-cell RNA-Seq, we found that MMR caused transcriptomic changes in CD14^+^ monocytes and NK cells, but most profoundly in γδ T cells. Monocyte function was not altered by MMR vaccination. In contrast, the function of γδ T cells was markedly enhanced by MMR vaccination, with higher production of TNF and IFN-γ, as well as upregulation of cellular metabolic pathways. In conclusion, we describe a trained immunity program characterized by modulation of γδ T cell function induced by MMR vaccination.

## Abstract 2

MMR vaccination induces cellular and metabolic reprogramming in &gamma;&delta; T cells, leading to a more active phenotype.

## Graphical abstract

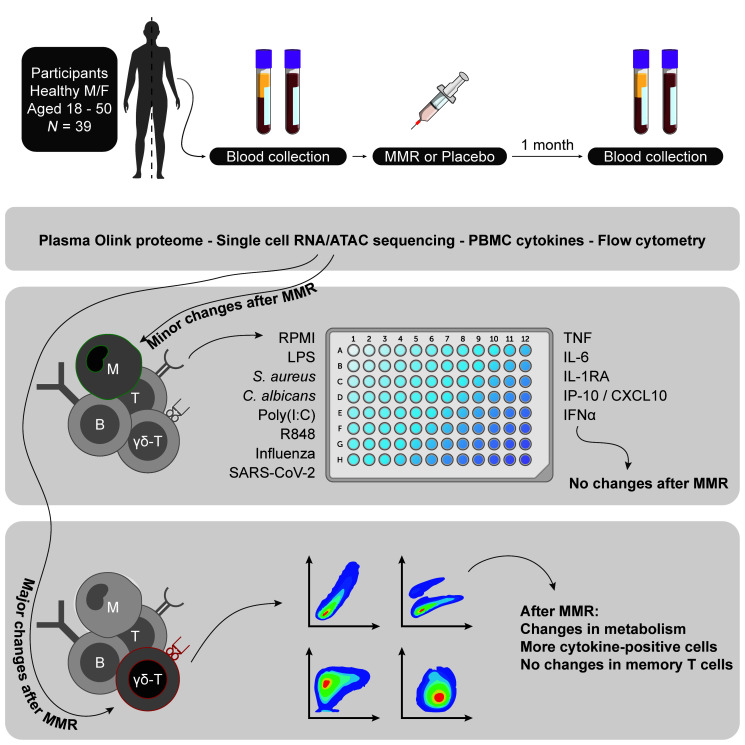

## Introduction

Vaccines are developed to target specific pathogens. However, an accumulating body of evidence suggests that certain live-attenuated vaccines provide a broad spectrum of protection against heterologous infections as well (nonspecific effects [NSEs]) (1, 2). Vaccine-induced NSEs are accompanied by epigenetic and metabolic changes in innate immune cells, an immunological process termed trained immunity that represents a de facto memory of innate immunity (3–6). Recent studies suggest that the induction of trained immunity is an attractive strategy to boost broad protection against various infections on the one hand, as well as improving anticancer immunotherapy on the other hand (7). Most of the studies aiming to study trained immunity induced by vaccines in humans have used the tuberculosis vaccine bacille Calmette-Guérin (BCG) (8–10), while very little is known about the effects of other vaccines on innate immune cells. Although there is ample epidemiological evidence that other live-attenuated vaccines also have NSEs, their potential effects on innate immune cells have not been studied.

Measles-containing vaccines (MCVs) are one such group of vaccines associated with beneficial heterologous effects (11). Measles vaccines are routinely used in childhood immunization programs worldwide and were recently reconfirmed to be safe and effective (12). The measles, mumps, and rubella (MMR) vaccine is composed of a live-attenuated, negative-stranded measles virus, combined with mumps and rubella, and provides lifelong immunity against measles after 2 doses. Several studies have confirmed higher survival and lower morbidity for children after measles immunization, independent of measles-attributable disease (13, 14). One recent study suggested that measles infection diminishes the protective antibodies against other infections (15), and the NSEs of MMR may be mediated by negating these immunosuppressive effects. In addition, it can be also envisaged that MMR induction of trained immunity can also contribute to the broad heterologous protection (16, 17). Here, we assess the potential of MMR vaccination to induce trained immunity against SARS-CoV-2 and a range of other microbial stimuli. We used single-cell RNA-Seq (scRNA-Seq) and single-cell assay for transposase-accessible chromatin using single-nucleus assay for transposase-accessible chromatin using sequencing (snATAC-Seq) to compare cellular heterogeneity in a randomized, placebo-controlled trial of MMR revaccination in Dutch adults. Interestingly, we found that MMR vaccination caused transcriptomic and functional changes in γδ T cells, rather than in monocytes. This suggests that γδ T cells might have a key role in the mechanisms underlying MMR-induced trained immunity and NSEs.

## Results

### Study design and baseline characteristics.

**Figure 1:**
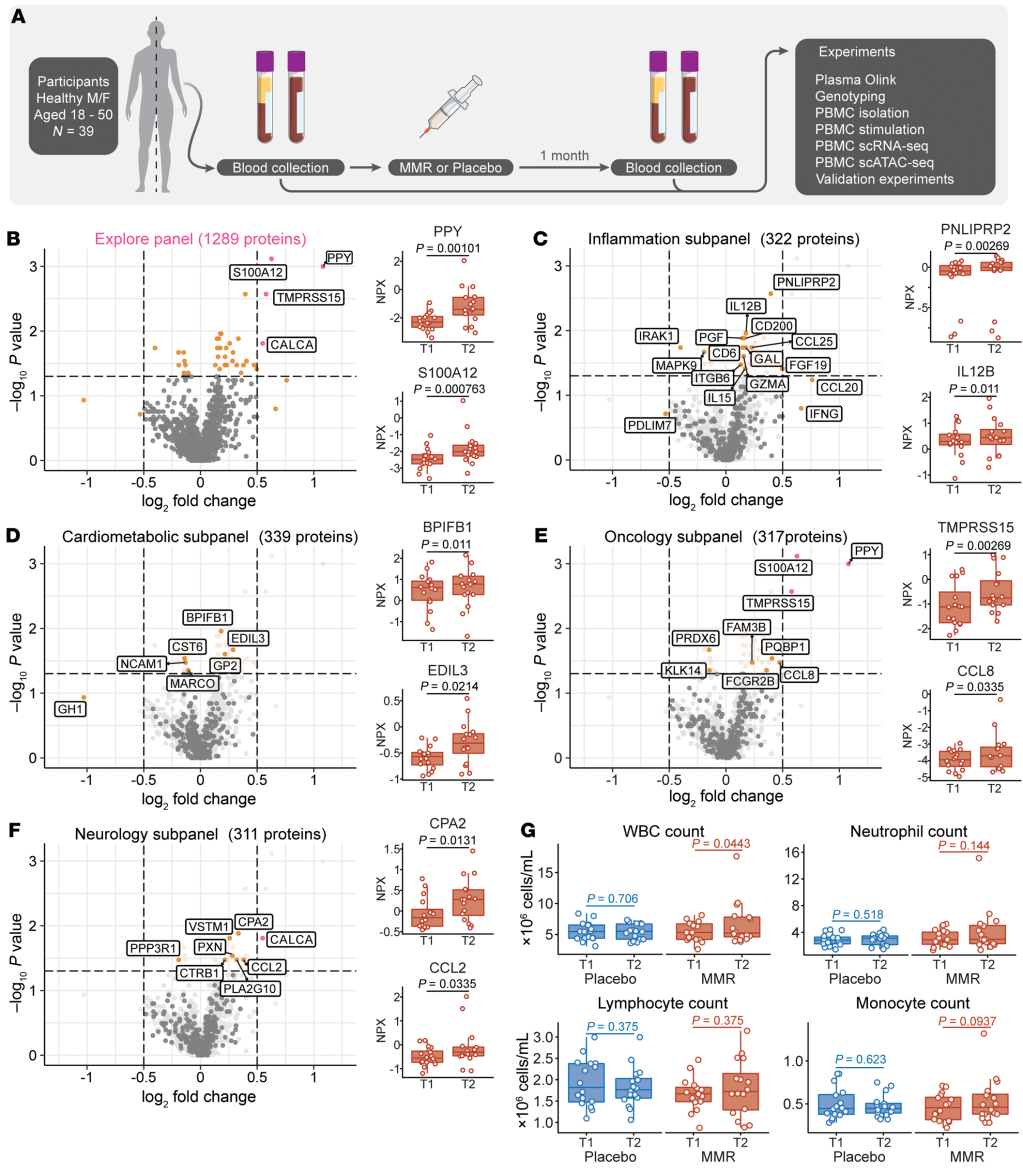
Study setup, plasma proteomics analysis after MMR vaccination, and WBC counts. (**A**) Setup of the present randomized, placebo-controlled trial of MMR vaccination. M/F, males/females. (**B**) Volcano plot of Olink targeted proteomics (*n* = 1,289 analyzed proteins in total) in plasma, after MMR vaccination (*n =* 16). (**C**–**F**) Volcano plots of subcategories of the plasma proteome measured by Olink. In the volcano plots for the subpanels, the full panel is depicted in light gray as the background. The side plots in panels **B**–**F** show the relative expression values (NPX) of selected proteins in each (sub)panel. (**G**) Total and differentiated WBC counts, before and after vaccination in placebo- and MMR-vaccinated groups. An unadjusted *P* value of less than 0.05 is the cutoff for the volcano plots. *P* values for this figure were calculated using the Wilcoxon signed-rank test. T1, baseline; T2, one month after treatment.

To investigate the potential nonspecific effects of MMR revaccination in adults, we conducted an exploratory randomized, controlled trial (Figure 1A; see Methods for more details). Briefly, 39 healthy adults (*n* = 19 women and *n* = 20 men) were randomly assigned to receive either the MMR vaccine or a placebo. All participants were between 18 and 50 years of age, and there were no significant differences in sex, age, or BMI between the vaccination arms (Supplemental Figure 1 and Supplemental Table 1; supplemental material available online with this article; https://doi.org/10.1172/JCI170848DS1). Peripheral venous blood was collected to conduct immunological analyses immediately before vaccination and 1 month later. No infections occurred in these individuals between visits or at least 2 weeks prior to the baseline measurement.

### Circulating biomarkers of inflammation after MMR vaccination.

Previous studies have shown that trained immunity induced by BCG increases the responsiveness of innate immune cells upon rechallenge but reduces systemic inflammation during homeostasis (8). We sought to determine whether this is also the case after MMR vaccination. To that end, we used Proximity Extension Assay technology (Olink) to assess targeted proteomics biomarkers (which have previously been related to inflammation, oncology, neurology, or cardiometabolic function) before and after MMR vaccination (Figure 1B and Supplemental Data Set 1). We found no major changes in plasma proteome composition between the baseline measurement and 1 month after vaccination. Of all analyzed parameters (1,289 after quality control [QC]), only 4 met our selected cutoffs for a log_2_ fold change of greater than 0.5 and an unadjusted *P* value of less than 0.05. These were PPY (a pancreatic protein associated with counterregulation of gastric emptying; ref. 18), S100A12 (a calcium-binding alarmin protein; ref. 19), TMPRSS15 (a peptidase known to activate trypsin; ref. 20), and CALCA (a vasodilating peptide hormone involved in calcium regulation and thought to also function as a neurotransmitter; ref. 21).

We subsequently assessed the proteins that met the statistical significance threshold, independently of the fold change (Figure 1, C–F). Of the protein subcategories, the inflammation-related proteins showed the highest number of changes, with a trend toward upregulation after vaccination (Figure 1C). PNLIPRP2, the top significant hit from this subpanel, is a pancreas-associated protein involved in lipid metabolism. Among the other enriched proteins were factors related to NK cell and T cell activities (IL-12B, IL-15, IFN-γ, CCL20, GZMA). Of the cardiometabolic-related proteins (Figure 1D), most suggestive hits were also related to immunological processes either directly (GP2, CST6, NCAM1, MARCO) or indirectly (GH1). The proteins significantly changed in the oncology panel (Figure 1E) were similarly enriched for immunologically relevant proteins (S100A12, PQBP1, CCL8, FCGR2B), with a trend toward upregulation after vaccination. Finally, in the neurology panel (Figure 1F), there was also an upregulation of immunology-related proteins such as CCL2, VSTM1, and PLA2G10. These results indicate that inflammation-related proteins tended to be upregulated in the circulation after MMR vaccination, although the effect size was limited. Of note, the results of our proteome analysis should be interpreted with caution, as correction for multiple testing was not feasible, given the limited sample size. Thus, while we do not have strong evidence for differential abundance of individual biomarkers, the subgroup of particularly inflammatory markers as a whole tended to be upregulated. This pattern differs markedly from the inhibitory effects exerted on systemic inflammation by BCG vaccination (8).

We then considered the effects of MMR vaccination on circulating leukocyte counts (Figure 1G). MMR vaccination, but not placebo treatment, significantly increased (*P =* 0.04) the number of circulating leukocytes after 1 month, although there was considerable interindividual variation. This change appeared to be driven mainly by an increase in myeloid cells (neutrophils and monocytes), although the comparison did not reach statistical significance for the individual cell types. Together, our results show that the MMR vaccine slightly increased systemic inflammation 1 month after vaccination.

### Transcriptome effects in monocytes and γδ T cells after MMR vaccination.

We decided to further investigate the effects of MMR vaccination on the composition and cellular states of circulating leukocytes. To that end, we performed snATAC-Seq and scRNA-Seq on PBMCs from a subset of participants. For snATAC-Seq, we selected 12 participants from both the placebo- and MMR-treated groups. We then selected half of those individuals for scRNA-Seq, taking care to balance male and female participants from both the MMR and placebo groups.

**Figure 2:**
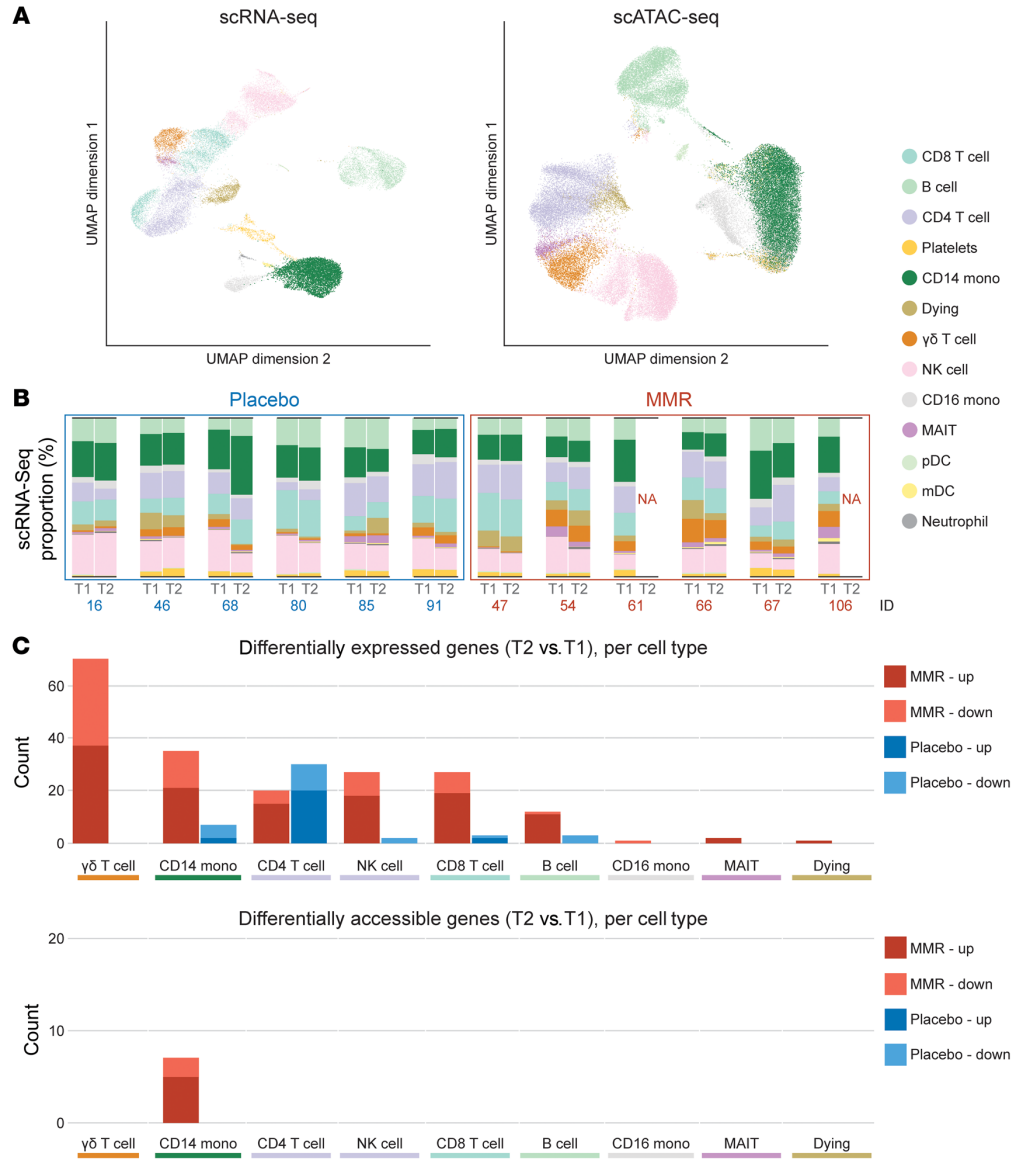
Single-cell analysis of PBMCs following MMR vaccination. (**A**) UMAP analysis of scRNA-Seq and snATAC-Seq of PBMCs. (**B**) Proportions of cell types annotated according to the scRNA-Seq data in placebo and MMR samples. (**C**) Top: Differentially expressed genes (scRNA seq) per cell type, between baseline (T1) and 1 month after placebo or MMR (T2). Bottom: differentially accessible genes (snATAC-Seq) per cell type, between baseline (T1) and 1 month after placebo or MMR (T2).

An integrated analysis of both scRNA-Seq and snATAC-Seq data (Figure 2A and Supplemental Figure 2, A and B) based on marker genes enabled us to identify 13 cell types within the PBMC fraction (Supplemental Figure 2C). Since the WBC differential (Figure 1G) suggested there might be changes in the PBMC composition, we leveraged the single-cell data sets to investigate this in more detail. Indeed, we observed intraindividual shifts in PBMC composition between time points (Figure 2B and Supplemental Figure 3A). However, we did not observe any consistent changes in either the MMR or placebo group (Figure 2B and Supplemental Figure 3, A and B). Although the low number of participants hindered statistical comparisons, our data suggest that it is unlikely that MMR vaccination has a major influence on PBMC composition 1 month after vaccination.

Previous studies have shown that vaccination with other live-attenuated vaccines such as BCG induces transcriptional and functional changes (trained immunity) in innate immune cells such as monocytes (22, 23). We therefore hypothesized that, although the PBMC composition was not altered by MMR vaccination, changes in monocyte transcriptional and functional programs could account for its known NSEs. We thus assessed the transcriptional effects of MMR and placebo between time points, across different cell types. In every cell type except CD4^+^ T cells, MMR vaccination had a more pronounced transcriptional effect than did placebo (Figure 2C, top panel). Indeed, transcriptional programs in CD14^+^ monocytes and NK cells, classical innate immune cell populations in which other vaccines (such as BCG) induce trained immunity, were prominently influenced by the MMR vaccine. Surprisingly, however, γδ T cells were the most strongly affected cells at the transcriptional level among all assessed cell types. Cell-type–specific analysis of differentially accessible genes revealed the chromatin to be affected by the MMR vaccine only in CD14^+^ monocytes (Figure 2C, bottom panel).

### MMR vaccination has minor effects on the transcriptome and epigenome of monocyte subpopulations.

**Figure 3:**
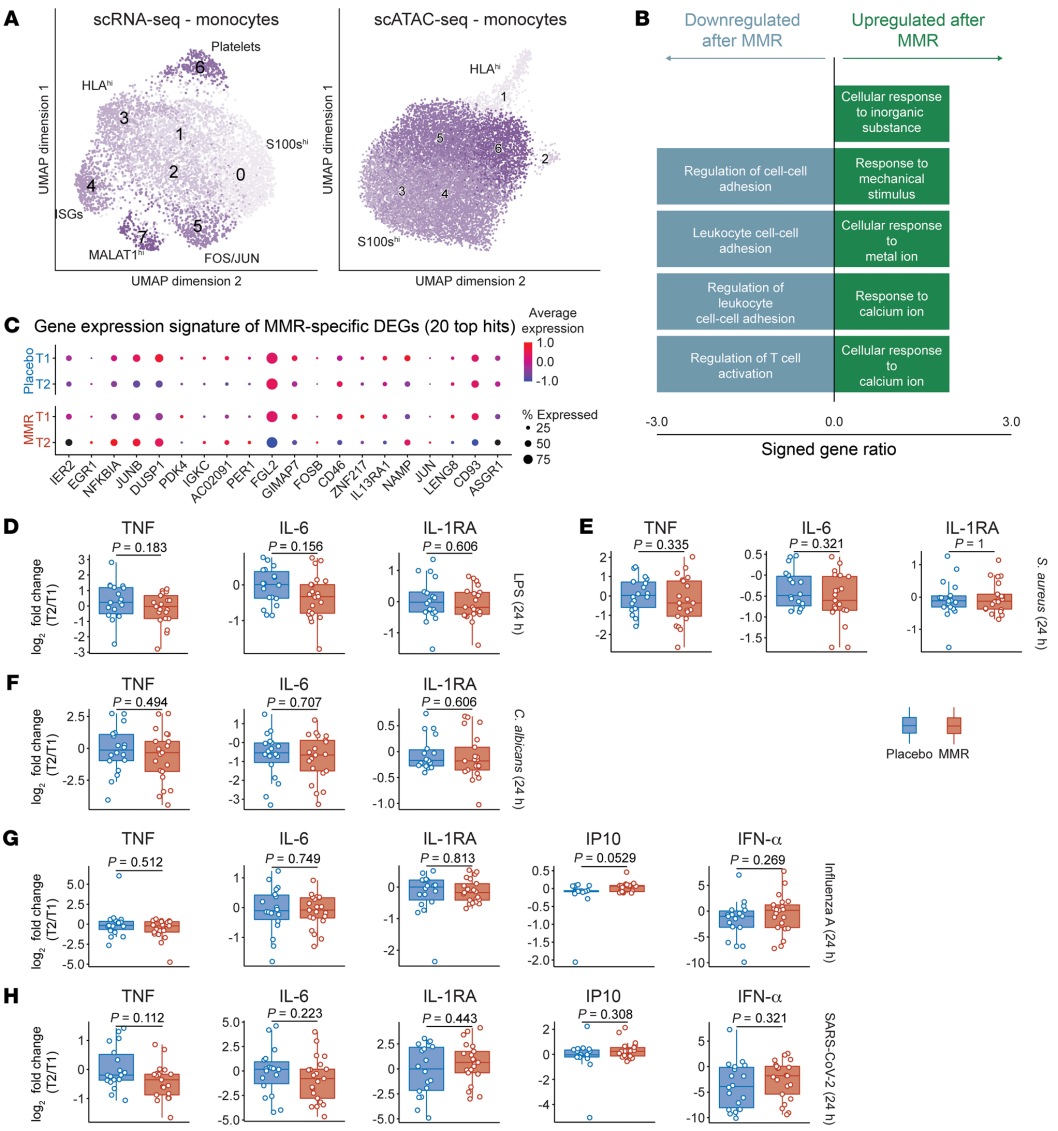
Single-cell analysis of monocyte subpopulations and monocyte-associated cytokine production by PBMCs. (**A**) UMAP analysis and subpopulation identification of scRNA-Seq and snATAC-Seq, specifically in monocytes. (**B**) Pathway enrichment of genes that were differentially expressed in monocytes after MMR vaccination. (**C**) Top 20 differentially expressed genes in monocytes following MMR vaccination, by time point and treatment group. (**D**–**H**) Monocyte-associated cytokines produced by PBMCs following diverse stimulations; the data are expressed as log_2_ fold changes between baseline and 1 month after treatment. *P* values for **D**–**H** were calculated using the Mann-Whitney *U* test. Box plots were drawn as follows: lower and upper hinges indicate the 25th and 75th percentiles; whiskers indicate the hinge plus 1.5 times the IQR; and the line in the box indicates the median.

Exploring further the monocyte sequencing data, we found different subpopulations defined by their transcriptional or open-chromatin signatures (Supplemental Data Set 2). Among them, we found a subpopulation of cells that highly expressed HLA genes and another cell subpopulation that upregulates alarmins, in both data layers (Figure 3A). We did not identify significant changes in the subpopulations in either data layer (Supplemental Figure 3A). Investigating the transcriptional changes induced by MMR vaccination, we identified more than 20 differentially expressed genes (adjusted *P* < 0.05) and 7 genes with changed chromatin accessibility (Supplemental Figure 4C). Upregulated genes were enriched in pathways associated with response to mechanical stimulation or exposure to metals such as calcium (*FOS*, *FOSB*, *JUN*, *JUNB*, and *NFKBIA*). Downregulated genes were enriched in cell-cell adhesion programs (*B2M*, *FGL2*, *CD46*, *PRKAR1A*) (Figure 3, B and C). Thus, while monocytes were moderately affected at the transcriptional level by MMR vaccination, these cells were not explicitly more proinflammatory during homeostasis.

### Cytokine production capacity after MMR vaccination.

Innate immune memory is defined as the long-term functional reprogramming of innate immune cells by a first stimulation, leading to an altered response toward restimulation. To investigate functional immune responses after MMR vaccination or placebo, we stimulated PBMCs for 24 hours with a variety of bacterial (LPS, heat-killed *Staphylococcus aureus*), fungal (heat-killed *Candida albicans*), and viral [poly(I:C), R848, influenza A (H1N1), SARS-CoV-2] stimuli and measured monocyte-associated cytokine responses by ELISA. We measured TNF, IL-6, and IL-1RA concentrations after stimulation, which are proinflammatory cytokines mainly produced by monocytes. Additionally, we measured IP10 and IFN-α specifically for the viral stimulations. We calculated log_2_-transformed fold changes corrected for age, sex, and BMI between 1 month after vaccination and baseline measurements, and statistically compared placebo treatment to MMR vaccination (Figure 3, B–H). We observed large intra- and interindividual variations in cytokine responses between baseline and 1 month after treatment. However, there were no significant differences between placebo and MMR for any measured cytokine across all stimuli. Thus, we found no differences in PBMC cytokine production capacity after MMR vaccination. As monocytes are the main producers of the measured cytokines in PBMCs during 24-hour stimulations, this suggests that monocyte cytokine secretion capacity is not changed by MMR vaccination.

### Transcriptional and functional reprogramming of Vδ2 T cells after MMR vaccination.

**Figure 4:**
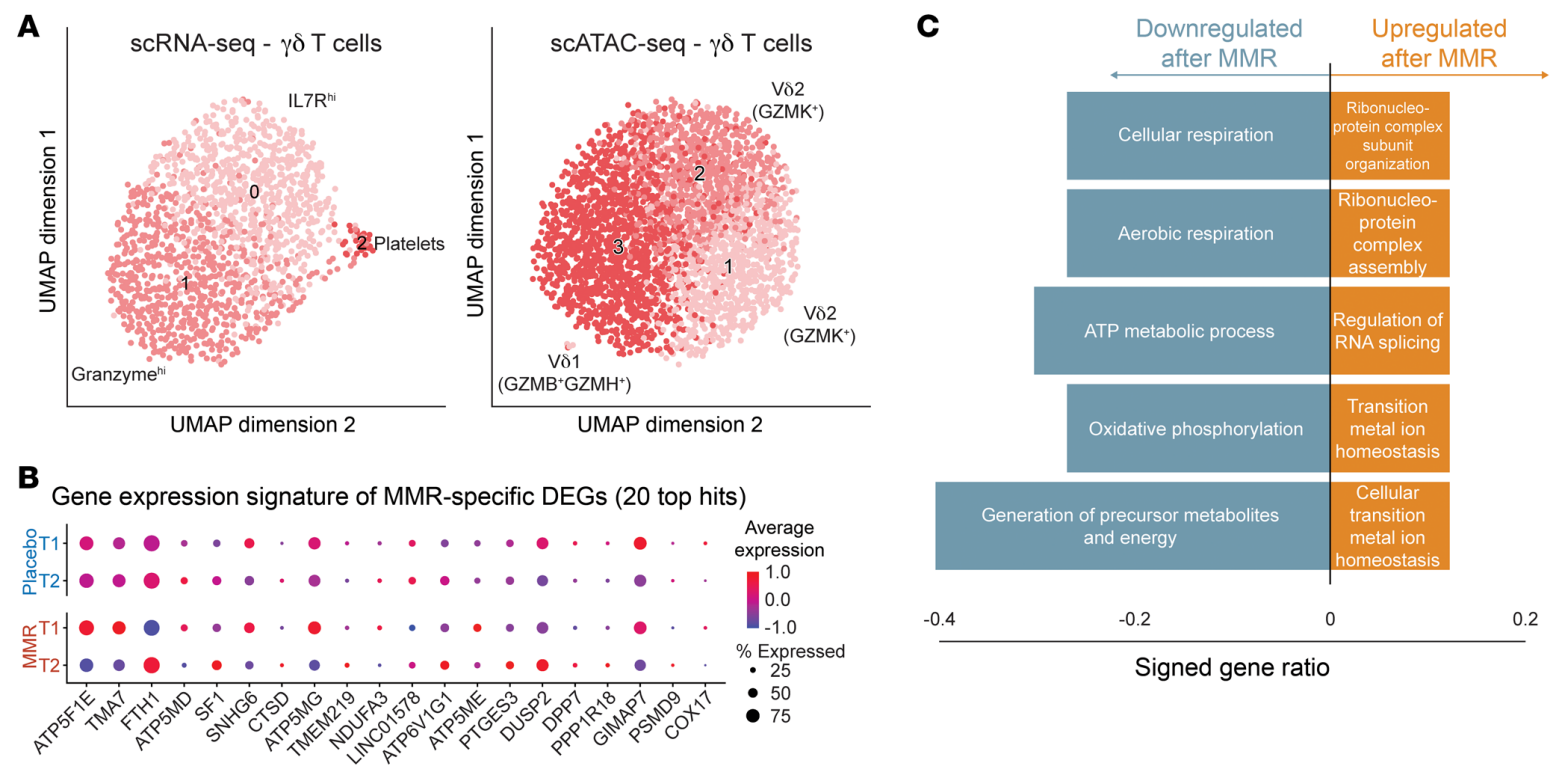
Single-cell analysis of γδ T cell populations. (**A**) UMAP analysis and subpopulation identification of scRNA-Seq and snATAC-Seq, specifically in γδ T cells. (**B**) Pathway enrichment analysis of genes that were differentially expressed in γδ T cells after MMR vaccination. (**C**) Top 20 differentially expressed genes in γδ T cells following MMR vaccination, by time point and treatment group.

Because the most prominent changes in response to MMR were seen in γδ T cells according to the scRNA-Seq data (Figure 2), in the next set of experiments we assessed whether vaccination changed the transcriptional and functional programs of these cells. A closer look into the γδ T cell subpopulations revealed differences in their transcriptional and open-chromatin dynamics (Supplemental Data Set 3). Transcriptionally, 2 distinct subpopulations were found, 1 characterized by transcription of granzyme genes and the other by upregulation of IL-7R (Figure 4A, left). Integrating open-chromatin landscape with their transcriptional profiles from the same individuals led to differentiating Vδ1 (higher in granzyme B [GZMB] and GZMH) and Vδ2 (higher in GZMK) T cell populations (Figure 4A, right). Notably, the vaccination did not affect the proportion of the subpopulations identified (Supplemental Figure 5, A and B). Interestingly, however, MMR induced a metabolic shift in γδ T cells at the transcriptional level, with downregulation of genes involved in cellular respiration and ATP synthesis (Figure 4, B and C), exemplified by *NDUFA3*, *ATP5F1E*, *ATP5MD*, and *ATP5MG*. We subsequently performed flow cytometry to assess the functional consequences for Vδ2 T cells (the most abundant γδ T cell population in the blood).

**Figure 5:**
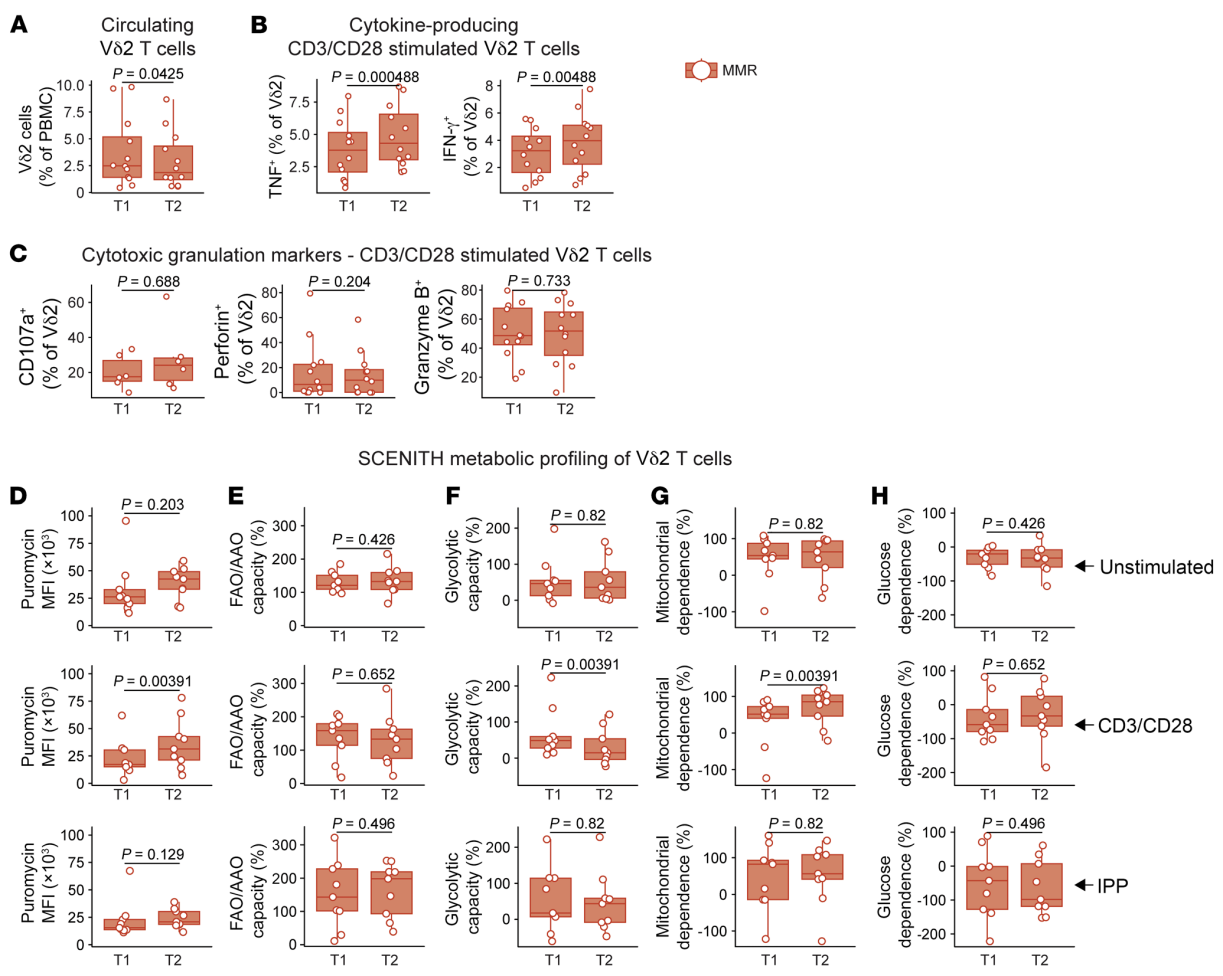
Functional and metabolic characterization of Vδ2 cells following MMR vaccination. (**A**) Percentage of Vδ2 T cells in isolated PBMCs. (**B**) Percentage of Vδ2 T cells that produced TNF or IFN-γ following CD3/CD28 stimulation. (**C**) Percentage of Vδ2 T cells expressing markers of cytotoxic granule release (CD107a) or production (perforin and GZMB). Metabolic parameters by modified SCENITH (https://www.scenith.com), calculated as in Argüello et al. (24): (**D**) puromycin incorporation, (**E**) fatty acid oxidation/amino acid oxidation (FAO/AAO) capacity, (**F**) glycolytic capacity, (**G**) mitochondrial dependence, and (**H**) glucose dependence. All parameters were measured by flow cytometry. *P* values were calculated using the Wilcoxon signed-rank test. Box plots were drawn as follows: lower and upper hinges indicate the 25th and 75th percentiles; whiskers indicate the hinge plus 1.5 times the IQR; and the line in the box indicates the median.

We noted a small but significant decrease in Vδ2 T cells present in the PBMC fraction of MMR-vaccinated individuals (*P =* 0.0425; Figure 5A). On the other hand, we did not find any significant differences in the relative abundance of subpopulations expressing CD27 and/or CD45RA, indicating that the MMR vaccine did not have strong effects on classical memory Vδ2 T cell formation (Supplemental Figure 6A). There were also no changes in the expression levels of CTLA4, PD1, LAG3, or TIM3, markers that are commonly associated with T cell exhaustion (Supplemental Figure 6D). Notably, following stimulation of the γδ T cell receptor using anti-CD3/anti-CD28 beads, the percentage (but not the MFI) of Vδ2 T cells positive for TNF or IFN-γ increased (Figure 5B and Supplemental Figure 6B). This indicates that the γδ T cell population had become more responsive toward secondary stimulation, a feature resembling the classical monocyte-trained immunity. Unstimulated Vδ2 T cells showed a trend toward lower expression of the degranulation marker CD107a after MMR vaccination (Supplemental Figure 6C); this effect was not present in CD3/CD28-stimulated Vδ2 T cells, which showed no difference between time points in the percentage of cells staining positive for CD107a, GZMB, or perforin (Figure 5C). These data suggest that granule release by Vδ2 cells was more tightly regulated at baseline following MMR vaccination but that this effector function was not weaker following stimulation.

The single-cell analyses revealed that unstimulated γδ T cells modulate genes associated with oxidative phosphorylation (Figure 4, B and C). Therefore, we determined the functional metabolism profile of Vδ2 T cells before and after MMR vaccination using SCENITH (24). This flow cytometry–based technique uses puromycin incorporation as a proxy for protein synthesis activity, which in itself reflects a substantial portion of total ATP used by the cell. Briefly, we measured protein synthesis levels (puromycin MFI, Figure 5D) upon treatment with metabolic inhibitors. This allowed us to the calculate fatty acid/amino acid oxidation capacity (Figure 5E), glycolytic capacity (Figure 5F), mitochondrial dependence (Figure 5G), and glucose dependence (Figure 5H) of Vδ2 T cells. Although the experiments revealed a trend toward higher protein synthesis activity in unstimulated Vδ2 T cells following MMR vaccination, this was not statistically significant (*P =* 0.203, Figure 5D, top). However, when Vδ2 T cells were stimulated with CD3/CD28, we noted a significant increase in protein synthesis (*P =* 0.00391, Figure 5D, middle). The same pattern was visible following stimulation with isopentenyl pyrophosphate (IPP), a common antigen specifically for Vδ2 T cells, although it did not reach statistical significance (*P =* 0.129, Figure 5D, bottom). Subsequently, in CD3/CD28-stimulated Vδ2 T cells, we observed a decrease in glycolytic capacity and thus a concomitant increased dependence on mitochondrial energy metabolism (Figure 5, F and G, middle panels). These differences were not significant for IPP stimulation (Figure 5, F and G, bottom panels).

The flow cytometric readouts for Vδ2 T cell function and metabolism were also performed on a limited number of placebo samples (*n =* 3 individuals, due to the limited availability of cryopreserved materials). These data are shown in Supplemental Figure 6, A–D, and Supplemental Figure 7, A–H, the latter being a placebo representation of Figure 5.

## Discussion

Trained immunity entails the process of boosting innate immune function following vaccination or infection (4), and this process has been proposed to mediate, at least in part, the heterologous protective effects of live-attenuated vaccines such as BCG and MMR. Although extensive studies have documented the induction of trained immunity by BCG (including but not limited to refs. 3, 5, 6, 8–10, 23, 25, 26), nothing is known regarding the capacity of MMR vaccination to induce trained immunity. We performed a randomized, placebo-controlled trial investigating the potential of the MMR vaccine to induce innate immune memory. Using single-cell multiomics transcriptional and epigenetic analysis combined with functional immunological and metabolic assays, we show that MMR vaccination induced a trained immunity phenotype in γδ T cells, whereas it had limited effects on monocyte function.

Most studies of the heterologous protection induced by certain vaccines such as BCG or influenza have focused on the induction of trained immunity in myeloid cells (3, 27). MMR vaccination has beneficial heterologous effects on overall mortality in children, therefore, we hypothesized that it also induces trained immunity through myeloid cells. We found that MMR induced modest changes in the chromatin accessibility and transcriptional programs of monocytes that are related to cellular responses to metal ions (specifically calcium, upregulated) and cellular adhesion (downregulated). However, these transcriptional effects did not result in significant effects on monocyte-derived cytokine production, despite the well-known role of calcium-dependent signaling in the function of immune cells (28–30). Future studies should be conducted to analyze in more depth the role of these pathways in monocytes after MMR vaccination and to determine whether these changes are functionally relevant.

Instead, we discovered that MMR induced much stronger transcriptomic and functional changes in the innate lymphoid population of γδ T cells. In this respect, MMR vaccination modulated the expression of genes involved in energy metabolism. We sought to functionally validate these findings and therefore closely examined the function of Vδ2 T cells, the most abundant γδ T cell subpopulation in human peripheral blood. MMR vaccination was followed by a significant increase in the proportion of Vδ2 T cells producing TNF (*P* = 0.000488) and IFN-γ (*P* = 0.00488), and these cells were more metabolically active, especially after CD3/CD28 stimulation. Our findings indicate that γδ T cells had a more activated phenotype in MMR-vaccinated individuals, providing a plausible mechanistic explanation for the NSEs conferred by MMR.

Because of their noncanonical antigen recognition mechanisms, the exact receptors and pathways that trigger the effects of MMR vaccination on the γδ T cell transcriptome and function remain to be investigated in future studies. Previous gene expression and functional analyses demonstrate that γδ T cells have hybrid innate and adaptive immune functions; the single-cell transcriptome of these cells has similarities to both CD8^+^ T cells and NK cells (31). Their innate-like features encompass the ability to mediate antibody-dependent cellular cytotoxicity, phagocytose pathogens, and direct rapid, nonspecific responses against threats (32). On the other hand, classic adaptive features of γδ T cells include somatic recombination of their functional T cell receptor, memory cell formation, and professional antigen-presenting capabilities (33). Unlike classical αβ T cell receptor signaling, antigen recognition by γδ T cells is not MHC restricted (34). For example, Vδ2 T cells predominantly recognize phosphoantigens such as IPP, (E)-4-hydroxy-3-methyl-but-2-enyl pyrophosphate (HMBPP) in the context of butyrophilins 2A1 and 3A1 (35), and tetanus toxoid (36, 37). In cancer, γδ T cells are known to exert strong antitumor effects by the release of proinflammatory cytokines, granzymes, and perforin and by activation of apoptosis-triggering receptors (37), although protumorigenic effects have also been described (38). Interestingly, previous studies have suggested that IFN-γ–producing γδ T cells are more dependent on glycolysis than on oxidative metabolism (39), whereas our analyses show an increased reliance on mitochondrial metabolism and, simultaneously, an increase in IFN-γ–producing Vδ2 T cells. This contrast may be explained by the differences between humans and mice, as well as between diseases (infections vs. cancer models) in the different studies. Future studies need to confirm our results and assess the full array of pathways and functional consequences induced by MMR on γδ T cells.

Our data have several practical and theoretical implications. On the one hand, the differences between the BCG-induced (myeloid-dependent) and MMR-induced (lymphoid-dependent) trained immunity programs demonstrate that different vaccines can induce different types of innate immune memory. In the future, this insight could aid in the development of a new generation of vaccines that could induce both adaptive and trained immunity–induced memory. This may involve incorporating synthetic ligands that activate trained immunity in γδ T cells to enhance either direct antipathogenic activities or indirect activation of other immune cells (such as CD8^+^ T cells or NK cells). Such approaches could improve nonspecific protection conferred by vaccines.

Specific to MMR, the demonstration that this vaccine was also able to induce trained immunity could lead to the hypothesis that it may have beneficial heterologous effects in groups of individuals such as the elderly and immunocompromised who have increased susceptibility to infections. Indeed, MMR had been proposed as a potential approach to prevent COVID-19 in the period before the SARS-CoV-2–specific vaccines were available (40, 41). In a case-control study during a recent measles outbreak, a reduced COVID-19 incidence was noted in men with MMR vaccination (42). One important aspect that remains to be investigated is related to the duration of the immunological effects of MMR vaccination on the function of γδ T cells. Prior research conducted in high-income countries has indicated that when children receive live vaccines, with MMR (administered at 15 months) being the most recent vaccine administered, they tend to experience fewer hospital admissions due to unrelated infections during their second year of life (13, 14, 17). This finding would indicate a potential immunological effect of up to 9 months of the MMR vaccine and is consistent with other live-attenuated vaccines such as BCG that are associated with the induction of trained immunity, but further study is needed.

Induction of trained immunity is not the only proposed mechanism to explain the heterologous effects of MMR on COVID-19. An inverse correlation between COVID-19 severity and MMR-specific IgG titers was found in adults in the United States (43). A potential explanation for this observation could lie in a cross-reactivity against structurally similar components of SARS-CoV-2 and MMR epitopes, which was described by Marakosova et al. (44, 45). However, this cannot account for the entire breadth of protection offered by MMR vaccines, and these studies did not investigate the potential effect of the MMR vaccine on innate immune cells.

Our study also has some limitations. First, the sample size was limited due to the exploratory nature of this investigation, which barred us from investigating the host and environmental factors that affect these MMR vaccination effects. Although MMR vaccination seemed to be associated with a slightly higher inflammatory proteomics signature, this does not necessarily apply at the level of individual biomarkers, since statistical testing with appropriate correction for multiple testing was precluded by the limited sample size. Future research should encompass an increased number of participants and a broader range of study parameters such as microbiome constituents, epigenetic histone modifications, and more follow-up time points, similar to the large-scale BCG vaccination studies (8, 26). Second, although NK-related inflammatory proteins were increased in the serum of MMR-vaccinated individuals, we could not substantiate this finding with functional NK cell experiments, given the limited number of available cells. Third, it is unknown which MMR components triggered γδ T cell activation, or if they reacted, for example, upon interaction with other activated cells. Moreover, in this study, we in fact revaccinated adults who had previously received the MMR vaccine as part of the Dutch national vaccination program. Future studies should investigate whether primary MMR vaccination has the same effect. Finally, in-depth assessments of the functional consequences and the mechanisms mediating γδ T cell activation were not possible, given the lack of an animal model of MMR vaccination, since mice, for example, do not express human CD46 and CD150, which act as receptors for the measles virus (and also, specifically, the Edmonston strain present in the MMR vaccine) (46).

In conclusion, our study shows, for the first time to our knowledge, that the MMR vaccine induces a program of trained immunity based on long-term transcriptional and functional changes in γδ T cells. The immunological and metabolic cellular responses to the MMR vaccine in our study revealed that γδ T cells represent a population of innate-like cells that mediate trained immunity. Our findings warrant further research to investigate the possibility that γδ T cell activation may be a component of trained immunity programs of other vaccines as well, and to assess the potential to improve vaccine efficacy by inducing these effects.

## Methods

### Sex as a biological variable

Our study examined male and female volunteers. We did not report sex-stratified analyses, as we did not have sufficient statistical power to do so in this exploratory study.

### Study participants

Thirty-nine healthy volunteers (Supplemental Table 1) of Dutch descent between the ages of 18 and 50 years were recruited between June and September 2020. Individuals with a medical history of immunodeficiency or a solid or nonsolid malignancy within the 2 preceding years were excluded. Vaccination 3 months prior to the start of the study or plans to receive other vaccinations during the study period was not allowed. Acute illness within 2 weeks of the study’s initiation or the use of drugs, including NSAIDs, less than 4 weeks before the start of the trial, with the exception of oral contraceptives, also resulted in exclusion. Pregnant individuals were not eligible.

### Blood collection and sample processing

EDTA whole blood (8 × 10 mL) was collected via venipuncture. Two of the EDTA tubes were centrifuged immediately after collection at 2,970*g* for 10 minutes at room temperature (RT), and plasma was stored at –80°C until later analysis. Hematological parameters such as WBC count and differential were measured on a Sysmex XN-450 apparatus. Cell counts were compared between time points using (unadjusted) Wilcoxon signed-rank tests. Additionally, 1 mL whole blood was stored at –80°C for genotype analysis.

PBMCs were isolated by density-gradient centrifugation over Ficoll-Paque (GE Healthcare). Briefly, EDTA blood was diluted in calcium/magnesium-free PBS and layered on Ficoll-Paque solution. After centrifugation for 30 minutes at 615*g* (no brakes, RT), the PBMC layer was collected and washed at least 3 times with cold calcium/magnesium-free PBS. The cells were resuspended in RPMI-1640, with Dutch modifications (Invitrogen, Thermo Fisher Scientific), and supplemented with 50 mg/mL gentamicin (Centrafarm), 2 mM GlutaMAX (Gibco, Thermo Fisher Scientific), and 1 mM pyruvate (Gibco, Thermo Fisher Scientific) and counted by Sysmex.

For cytokine production assessments, PBMCs were seeded in round-bottomed, 96-well plates at 0.5 × 10^6^ cells/well. The cells were stimulated for 24 hours using the stimuli described in Supplemental Table 2 (all in the presence of 10% pooled human serum, at 37°C and 5% CO_2_). Supernatants were collected and stored at –20°C until further analysis.

### PBMC freezing and thawing

Leftover PBMCs were resuspended in ice-cold, heat-inactivated FBS prior to cryopreservation. Ice-cold 20% DMSO in FBS was added dropwise to the cells until a final concentration of 10% DMSO was reached. The cells were stored for up to 24 hours in CoolCell alcohol-free freezing containers (Corning) at –80°C, after which they were transferred to a –150°C freezer for long-term storage. For subsequent experiments, PBMCs were thawed following a protocol modified from Hønge et al. (47). The PBMCs were retrieved from the –150°C storage and kept on dry ice until the moment of thawing. The cells were rapidly warmed in a water bath of 37°C until only a small clump of ice was present in the vial. The contents were immediately transferred into a 10× volume of prewarmed thawing medium (RPMI supplemented as described above and further supplemented with 20% FBS and 12.5 μg/mL DNase-I). The cells were centrifuged at 500*g* for 10 minutes at RT and resuspended in thawing medium without DNase-I. The cells were again centrifuged, resuspended in cold PBS, and counted with trypan blue to assess recovery and viability.

### ELISA cytokine measurements and data analysis

The cytokines TNF (commonly referred to as TNF-α), IL-6, IL-1Ra, and IP-10 were measured using DuoSet ELISA kits from R&D Systems, and IFN-α was measured with a kit from PBL Assay Science, according to the manufacturer’s protocol. To account for plate-to-plate variation, the participants were randomized over different plates (time points were kept together on the same plate). Cytokine concentrations were calculated relative to the standard curve in Gen5 software (BioTek). We calculated log_2_ fold changes between T2 and T1 and corrected for sex, age, and BMI using a linear regression approach. The MMR and placebo groups were compared using Mann-Whitney *U* tests.

### Targeted proteomics analysis by proximity extension assay

Plasma samples from 16 MMR-vaccinated individuals were sent to Olink (Sweden) for targeted proteomics analysis using proximity extension assay technology. In total, 1,472 proteins were measured, 183 of which were removed from the analysis because they were poorly detectable in more than 25% of the samples (30 cardiometabolic proteins, 46 inflammatory proteins, 56 neurology proteins, 51 oncology proteins). Unadjusted *P* values were calculated using the Wilcoxon signed-rank test.

### DNA isolation and genotyping

Whole blood samples were shipped on dry ice to the Human Genomics Facility of the Genetic Laboratory of the Department of Internal Medicine at Erasmus MC (Rotterdam, Netherlands). There, DNA isolation was performed and samples were genotyped using the Illumina GSA Arrays Infinium iSelect 24 × 1 HTS Custom Beadchip Kit.

### Single-cell library preparation and sequencing

Cryopreserved PBMCs were thawed as described above and washed an additional time with ice-cold PBS. Single-cell gene expression libraries were generated on the 10X Genomics Chromium platform using the Chromium Next GEM Single Cell 3′ Library and Gel Bead Kit, version 3.1, and the Chromium Next GEM Chip G Single Cell Kit (10X Genomics) according to the manufacturer’s protocol. snATAC-Seq libraries were generated on the 10X Genomics Chromium platform using the Chromium Next GEM Single Cell ATAC Library and the Gel Bead Kit, version 1.1, and the Chromium Next GEM Chip H Single Cell Kit according to the manufacturer’s protocol. Gene expression and ATAC-Seq libraries were sequenced on a NovaSeq 6000 S4 flow cell using version 1.5 chemistry (Illumina).

### Single-cell sequencing data analysis

#### Preprocessing and demultiplexing scRNA-Seq and snATAC-Seq data.

The proprietary 10X Genomics CellRanger pipeline (version 4.0.0) was used with default parameters. CellRanger count or CellRanger-atac count was used to align read data to the reference genome provided by 10X Genomics. refdata-cellranger-arc-GRCh38-2020-A-2.0.0 was used for the snATAC-Seq experiments and refdata-gex-GRCh38-2020-A for the scRNA-Seq. In scRNA-Seq, a digital gene expression matrix was generated to record the number of unique molecular identifiers (UMIs) for each gene in each cell. In snATAC-Seq, fragment files were created.

Each library was further demultiplexed by assigning cell barcodes to their donor. Souporcell (version 1.3 gb) (48) was used for genotype-free demultiplexing by calling candidate variants on the premapped bam files. Cells were clustered by their allelic information, and each cluster was matched to a donor with a known genotype.

#### scRNA-Seq data analysis.

The expression matrix from each library was loaded into the R/Seurat package (version 3.2.2) (49) for downstream analysis. To control the data quality, we first excluded cells with ambiguous assignments from the Souporcell demultiplex. Next, we further excluded low-quality cells with more than 25% mitochondrial reads, fewer than 100 or more than 3,000 expressed genes, or more than 5,000 UMI counts.

After QC, we applied logNormalization (Seurat function) and scaled the data, regressing for total UMI counts, the number of features, the percentage of mitochondrial genes, the and percentage of ribosomal genes. We then performed principal component analysis (PCA) based on the 2,000 more highly variable features identified using the vst method implemented in Seurat. As batches showed a good integration of the data, no integration algorithm was applied. Cells were then clustered using the Louvain algorithm with a resolution of 0.75 based on neighbors calculated in the first 30 principal components. For visualization, we applied uniform manifold approximation and projection (UMAP) based on the first 30 principal components. For clarity, 2 samples were excluded from the analysis on the basis of the QC results (see Figure 2B).

#### Annotation of scRNA-Seq clusters.

Clusters were annotated by manually checking the expression of known marker genes. Cluster 5 showed higher expression of γ and δ chain genes (*TRGC1*, *TRDC*) along with T cell markers (*CD3E*). Dimensionality reduction and clustering analysis of this subset revealed 2 mixed cell populations: mucosal-associated invariant T cells (MAIT cells) overexpressing *KLRB1* and γδ T cells overexpressing *TRDC* and *TRGC*; these cells were then annotated accordingly.

#### Differential gene expression and gene set enrichment.

For paired comparison between time points in both MMR and placebo groups, differential expression (DE) tests were performed using the FindMarkers functions in Seurat with MAST (50). Patient IDs were regressed out in order to perform a paired analysis. Genes with a Bonferroni-corrected *P* value of less than 0.05 were considered to be differentially expressed.

Gene set enrichment was performed using the enrichGO function in the R package clusterProfiler. Gene sets enriched with a Benjamin-Hochberg–corrected *P* value below 0.05 and more than 4 genes were considered significant.

#### snATAC-Seq data analysis.

ArchR (51) was used for the downstream analyses of snATAC-Seq data,with reading of the fragment files created in by CellRanger-atac. Cells with fewer than 1,000 unique fragments, a transcription start site (TSS) enrichment below 4, identified as doublets by ArchR, or ambiguously labeled by souporcell were removed. To improve cell-type annotation of the snATAC-Seq data, a cell cluster that contained a significantly lower TSS enrichment and was not closely clustering to the well-separated cell-type clusters in the UMAP was removed.

After QC, the ArchR function “addIterativeLSI” was used to process iterative latent semantic indexing using the top 25,000 variable features and the top 30 dimensions. For visualization, UMAP with nNeighbors = 30 and minDist = 0.5 was applied.

Gene scores were calculated for each cell on the basis of accessibility. In order to aid the analysis of γδ T cells, a modified reference was used. By adding the gtf gene reference used by CellRanger, gene scores could be calculated for *TRDC*, *TRGC1*, and *TRGC2*.

#### snATAC-Seq annotation and integration with scRNA-Seq data.

ArchR function “addGeneIntegrationMatrix” was used to compare the calculated snATAC-Seq gene score matrix and the gene expression levels in scRNA-Seq data. This resulted in a matched scRNA profile and predicted the cell type per sequenced cell in the snATAC-Seq data. Cell types were therefore assigned to the snATAC-Seq data on the basis of the predicted cell type of the integration. UMAPs of the integrated blocks were inspected in order to examine the quality of the integration (Supplemental Figure 2, A and B).

#### Per-cell-type analysis of snATAC-Seq data.

A common approach was followed to inspect the open-chromatin changes in each cell type. The same method as described before for the whole-cell pool was used for visualization and clustering separately in each cell type. After this, open-chromatin peaks were calculated by running “addReproduciblePeakSet” using Macs2 algorithm (52). Transcription factor motif deviations were calculated on the basis of “CIS-BP” database annotation (53) using the “addDeviationsMatrix” function. The effects of MMR vaccination and placebo were assessed by running “getMarkerFeatures,” comparing both time points for all data types: open-chromatin peaks, TF motifs, and gene score. A FDR of less than 0.05 was indicative of significant changes.

### Flow cytometric measurements of γδ T cell parameters

Flow cytometric staining was performed as follows: 5 × 10^5^ thawed PBMCs were stained for surface markers using the antibodies described in Supplemental Table 3, for 30 minutes in the dark at 4°C, in FACS buffer (PBS, 5% FBS, 2 mM EDTA Intracellular proteins were analyzed after fixation and permeabilization in Cytofix permeabilization/fixation reagent (BD Biosciences) for 30 minutes. Following 2 washes with Cytofix permeabilization/washing buffer (BD Biosciences), cells were stained with the antibodies against the intracellular markers detailed in Supplemental Table 3, for 30 minutes in the dark at 4°C. After completion of the staining procedure, the cells were washed with PBS and stored in CellFIX reagent (BD Biosciences) until acquisition on a LSR II cytometer (BD Biosciences).

The flow cytometric data were analyzed in FlowJo (version X.07). The gating strategy was as follows: events corresponding to lymphocyte size were selected on the basis of forward scatter area/side scatter area (FSC-A/SSC-A), followed by selection of single-cell events in subsequent forward scatter height/forward scatter area (FSC-H/FSC-A) and forward scatter width/forward scatter area (FSC-W/FSC-A) gate. Viable cells were selected by gating on viability dye–negative cells. The analyses were performed on CD45^+^CD3^+^Vδ2^+^ cells. A visual example of this gating strategy is included in Supplemental Figure 8.

For the measurement of surface markers on unstimulated Vδ2 T cells, thawed PBMCs were stained as described above immediately after thawing. For measurements of cytokine expression or surface markers after stimulation, PBMCs were first treated with soluble anti-CD3/anti-C28 (BD Biosciences) antibodies for 4 hours in the presence of a Golgi plug (brefeldin A, BD Biosciences), under standard cell culture conditions.

Statistical comparisons between time points for all flow cytometric analyses (including SCENITH) were done using (unadjusted) Wilcoxon signed-rank tests.

### SCENITH methodology

We modified the original SCENITH technique (https://www.scenith.com) to analyze energy metabolism of γδ T cells. Briefly, PBMCs were plated at 0.3 × 10^6^ cells/well in 96-well plates. The cells were cultured in RPMI alone or stimulated with soluble anti-CD3/anti-CD28 antibodies or IPP for 4 hours under standard cell culture conditions. Next, cells were either left untreated (control) or were treated with 20deoxy-d-glucose (2-DG) (final concentration, 100 mM), oligomycin (O) (final concentration, 10 μM), and a combination of 2-DG and oligomycin (DGO) (final concentration 100 mM and 10 μM) for 30 minutes under standard cell culture conditions. Following the addition of puromycin (final concentration 10 μg/mL), the cells were incubated for an additional 45 minutes, and the cells were subsequently harvested and washed in cold FACS buffer before being stained as described above.

### Statistics

All data were analyzed in R as described in each relevant section of the Methods and figure legends. For paired comparisons (before-after vaccination), Wilcoxon signed-rank tests were used. For unpaired comparisons (between MMR and placebo groups), Mann-Whitney *U* tests were used. Statistical testing for differential gene expression/chromatin accessibility was performed using built-in statistical functions of the software packages as described above. Unless otherwise indicated, 2-tailed *P* values of less than 0.05 were considered statistically significant. If correction for multiple testing was applied, the method is described in the relevant Methods section. In cases where the *P* value is not provided, an asterisk indicates statistical significance. Box plots were drawn as follows: lower and upper hinges indicate the 25th and 75th percentiles, whiskers indicate the hinge plus 1.5 times the IQR, and the line in the box indicates the median.

The following R packages were used for the present work: the Tidyverse core packages 1.3.2 (54), Seurat 4.1.1, ArchR 1.0.2, SeuratObject 4.1.0, GenomicRanges 1.48.0, data.Table 1.14.2, ggplot2 3.3.6, colortools 0.1.6, clusterProfiler 4.4.4. magrittr 2.0.3, ggprism 1.0.3, ggsci 2.9, rstatix 0.7.0, pzfx 0.3.0, janitor 2.1.0, readr 2.1.3, openxlsx 4.2.5, psych 2.2.9. The figures were compiled using Adobe Illustrator.

### Study design, approval, and registration

This randomized, placebo-controlled trial, depicted in Figure 1A, was designed to research the ability of MMR vaccination to establish trained immunity. Therefore, participants were allocated 1:1 to receive either a placebo vaccine (0.1 mL 0.9% saline solution) or an MMR vaccine (SD, 0.5 mL, live-attenuated mumps virus [strain ‘Jeryl Lynn’, at least 12.5 × 10^3^ CCID50]; live-attenuated measles virus [strain ‘Enders’ Edmonston’, at least 1 × 10^3^ CCID50]; live-attenuated rubella virus [strain ‘Wistar RA 27/3’, at least 1 × 10^3^ CCID50]). Vaccination was performed intramuscularly in the right upper arm, and blood was drawn at baseline (T1) and 1 month after vaccination (T2). The trial protocol was approved in 2020 by the competent authority in the Netherlands and the Arnhem-Nijmegen Ethics Committee and was registered before participant recruitment in the following locations: (a) in the registry of the competent authority in the Netherlands (https://www.toetsingonline.nl/to/ccmo_search.nsf/), registration ID NL74082.091.20; (b) in the Dutch national trial registry (https://clinicaltrialregister.nl/), registration ID NL8609; and (c) in the European Union Drug Regulating Authorities Clinical Trials database (EudraCT; https://www.clinicaltrialsregister.eu/), registration ID EUCTR2020-002456-21-NL (the trial protocol can be accessed here).

The last 2 of these registrations are also accessible via the International Clinical Trials Registry Platform (https://trialsearch.who.int/). All experiments were conducted in accordance with the Declaration of Helsinki, and no adverse events were recorded. All participants provided written informed consent prior to inclusion in the study.

### Data and materials availability

The sequencing data used in this manuscript are accessible in the European Genome-Phenome Archive (EGA) (EGAS00001006787). The Olink data have been added as a supplemental file (Supplemental Data Set 1). A Supporting Data Values file containing all values for the data points in graphs and values behind any reported means is available in the supplemental materials. Other data are available upon request to the corresponding author.

## Author contributions 

RJR, PAD, and JBB contributed equally to this work, and each has the right to list themselves first in author order on their curricula vitae. MGN, YL, RJR, PAD, JBB, and KP conceptualized the study. PAD, RJR, JBB, OB, GK performed data curation. JBB, RJR, PAD, OB, GK, and VACMK conducted formal analyses. YL, MGN, and RJR acquired funding. PAD, RJR, TKS, OB, GK, AS, HB, HD, HL, EJDM, and YA performed experiments. PAD, RJR, JBB, SK, YL, KP, and MGN designed the study methodology. PAD, RJR, JTO, and MGN handled project administration. RJR, PAD, JBB, KP, EJDM, KLG, NR, PNO, LM, HS, OA, AB, YA, SK, YL, and MGN provided resources. JBB, RJR, PAD, OB, VACMK, and YL provided software. YL and MGN supervised the study. RJR, JBB, TKS, and KP verified data. RJR and JBB performed visualization. RJR, PAD, JBB, and MGN wrote the original draft of the manuscript. RJR, PAD, JBB, MGN, KP, JTO, HB, KS, YL, GK, and OB reviewed and edited the manuscript.

## Supplementary Material

Supplemental data

Supplemental data set 1

Supplemental data set 2

Supplemental data set 3

Supporting data values

## Version 1 01/30/2024

In-Press Preview

## Version 2 04/01/2024

Electronic publication
