# Supplementary material for: MMR vaccination induces trained immunity via functional and metabolic reprogramming of γδ T cells
Source: J Clin Invest. 2024 Jan 30;134(7):e170848. doi: 10.1172/JCI170848 (PMC10977989; doi:10.1172/JCI170848)
Supplement: Supplemental data [file jci-134-170848-s187.pdf]

Supplementary materials for:

MMR vaccination induces a trained immunity program characterized by functional and metabolic reprogramming of γδ T cells

Rutger J. Röring[1,2†], Priya A. Debisarun[1,2†], Javier Botey-Bataller[1,2,3,4†], Tsz Kin Suen[5], Özlem Bulut[1,2], Gizem Kilic[1,2], Valerie A. C. M. Koeken[1,2,3,4], Andrei Sarlea[1], Harsh Bahrar[1,2], Helga Dijkstra[1,2], Heidi Lemmers[1,2], Katharina L. Gössling[6], Nadine Rüchel[6], Philipp N. Ostermann[7], Lisa Müller[7], Heiner Schaal[7], Ortwin Adams[7], Arndt Borkhardt[6], Yavuz Ariyurek[8], Emile J. de Meijer[8], Susan Kloet[8], Jaap ten Oever[1,2], Katarzyna Placek[4], Yang Li[1,2,3,4], Mihai G. Netea[1,2,5]*

[†] These authors contributed equally to this work.

*Corresponding author:

Mihai G. Netea, MD, PhD

Department of Internal Medicine, Radboud University Nijmegen Medical Center

Tel: +31-24-3618819

E-mail: mihai.netea@radboudumc.nl

This PDF file includes:
Figs. S1 to S6
Tables S1 to S3

Other Supplementary Materials for this manuscript include the following:
Data S1 (Olink before and after MMR)

Supplementary figures

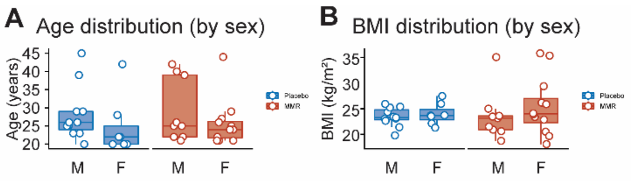

Figure S1: Participant characteristics. (**A**) Participant age, stratified by sex and treatment group. (**B**) Participant BMI, stratified by sex and treatment group.

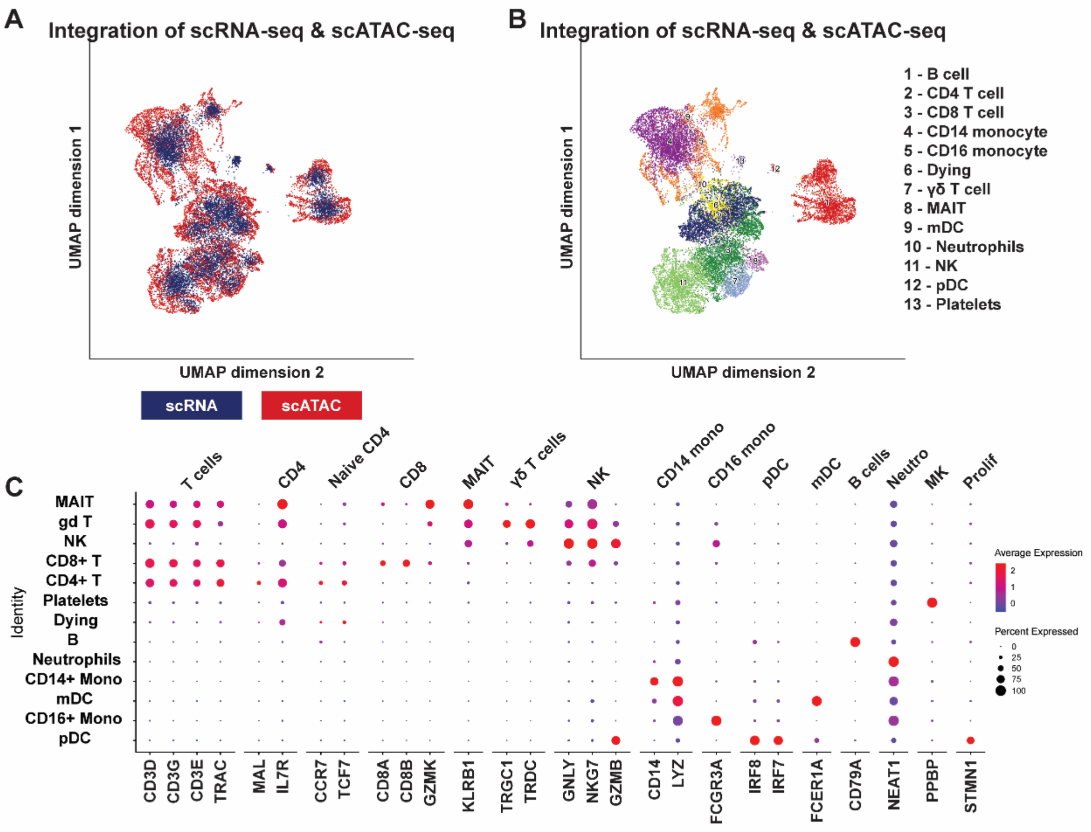

Figure S2: Integration of scRNA and scATAC-sequencing data and cell-type annotation. (**A**) UMAP of scRNA-seq and scATACseq integrated data. Integration was performed using canonical correlation analysis between gene expression values and gene score values calculated based on gene accessibility. ( ) Same as before, colored by the different celltypes present. (**C**) Dotplot of markers used for celltype annotation.

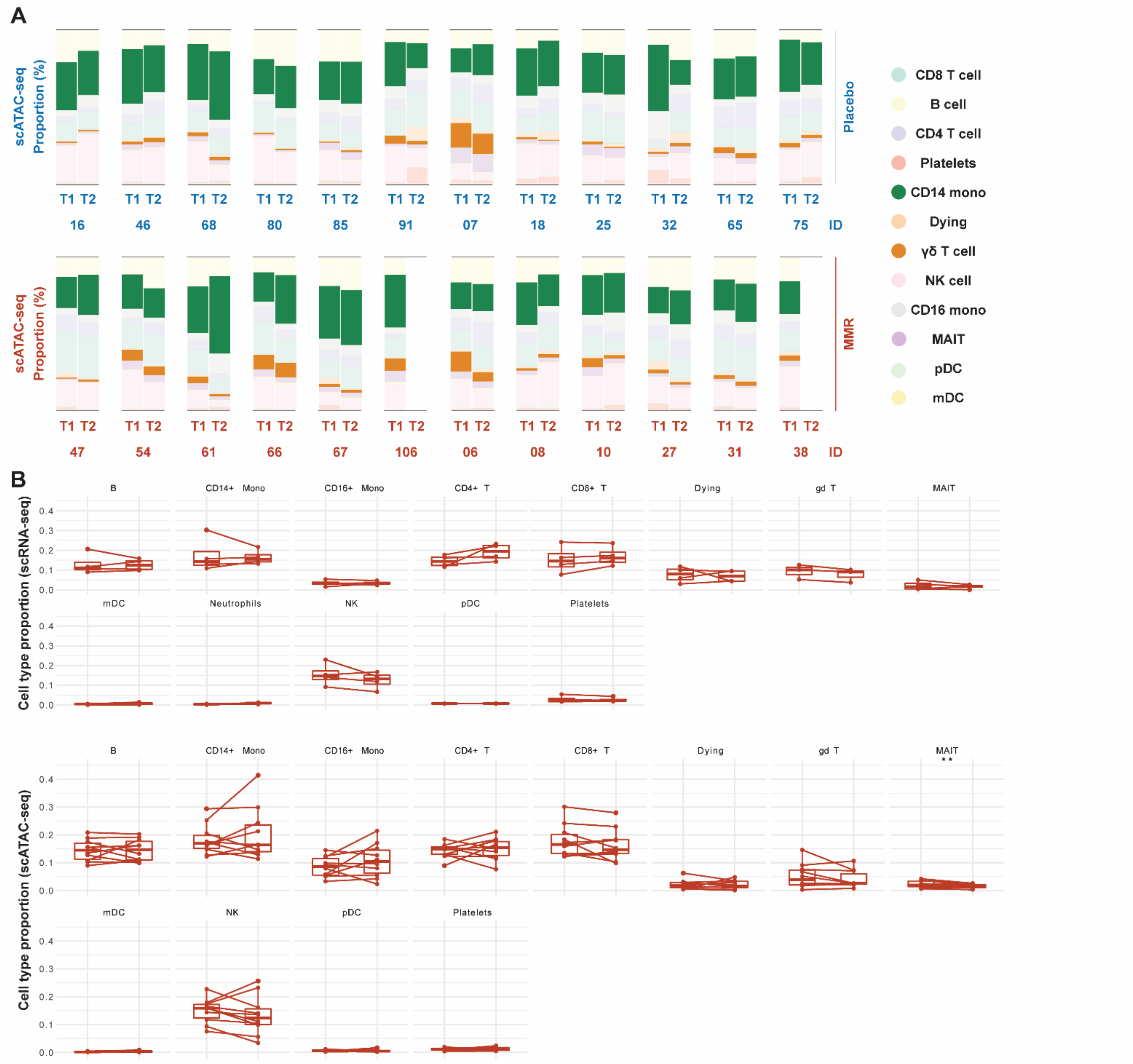

Figure S3: Cell-type proportions before and after treatment. (**A**) Proportions of cell types annotated according to the scATAC-seq data in placebo and MMR samples. (**B**) The same data, only displayed as boxplots per cell type to enable paired comparisons.

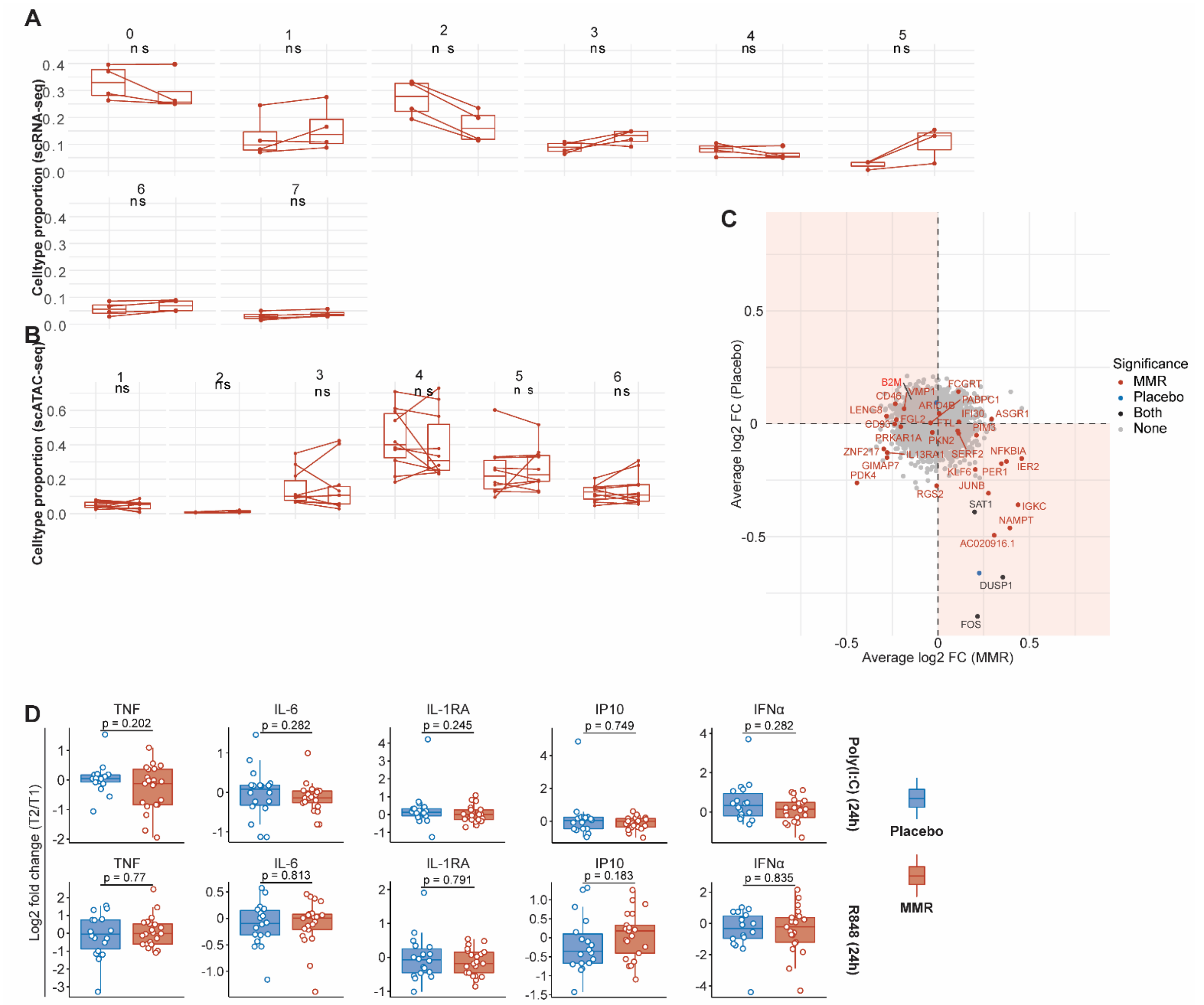

Figure S4: Single-cell analysis of monocyte subpopulations and monocyte-associated cytokine production by PBMCs. (**A**) Proportions of monocyte sub-populations (scRNA-seq) before and after MMR vaccination. (**B**) Proportions of monocyte sub-populations (scATAC-seq) before and after MMR vaccination. (**C**) Combined ‘volcano plot’ showing average log2 fold changes of monocyte gene expression between timepoints for both placebo and MMR. Bonferroni adjusted p-value < 0.05, paired test using MAST. (**D**) Monocyte-associated cytokines produced by PBMCs following diverse stimulations; the data are expressed as log2 fold-changes between baseline and one month after treatment.

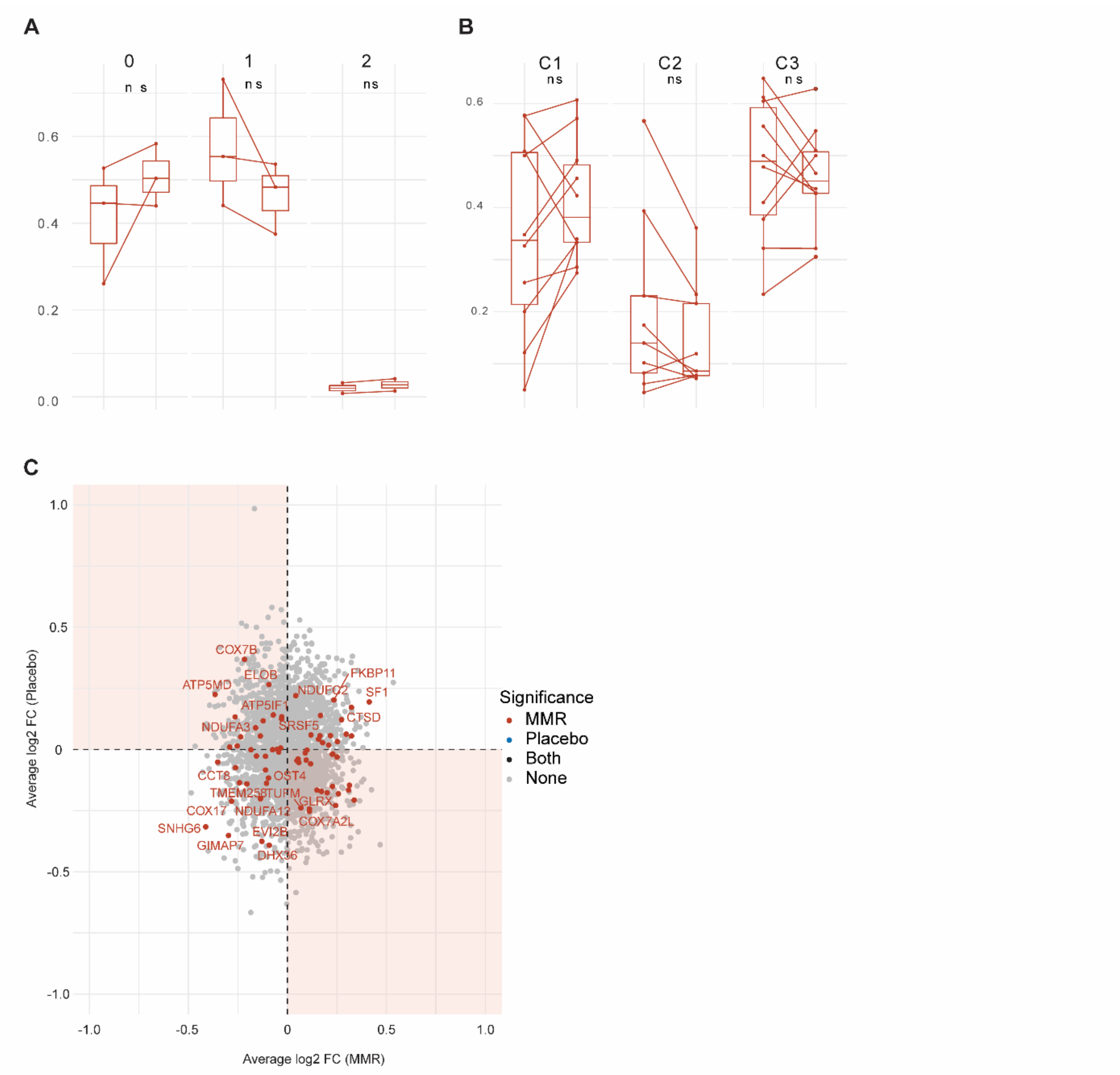

Figure S5: Single-cell analysis of γδ T cell populations. (**A**) Proportions of γδ T cell sub-populations (scRNA-seq) before and after MMR vaccination. (**B**) Proportions of γδ T cell sub-populations (scATAC-seq) before and after MMR vaccination. (**C**) Combined 'volcano plot' showing average log2 fold changes of γδ T cell gene expression between timepoints for both placebo and MMR. Bonferroni adjusted p-value < 0.05, paired test using MAST.

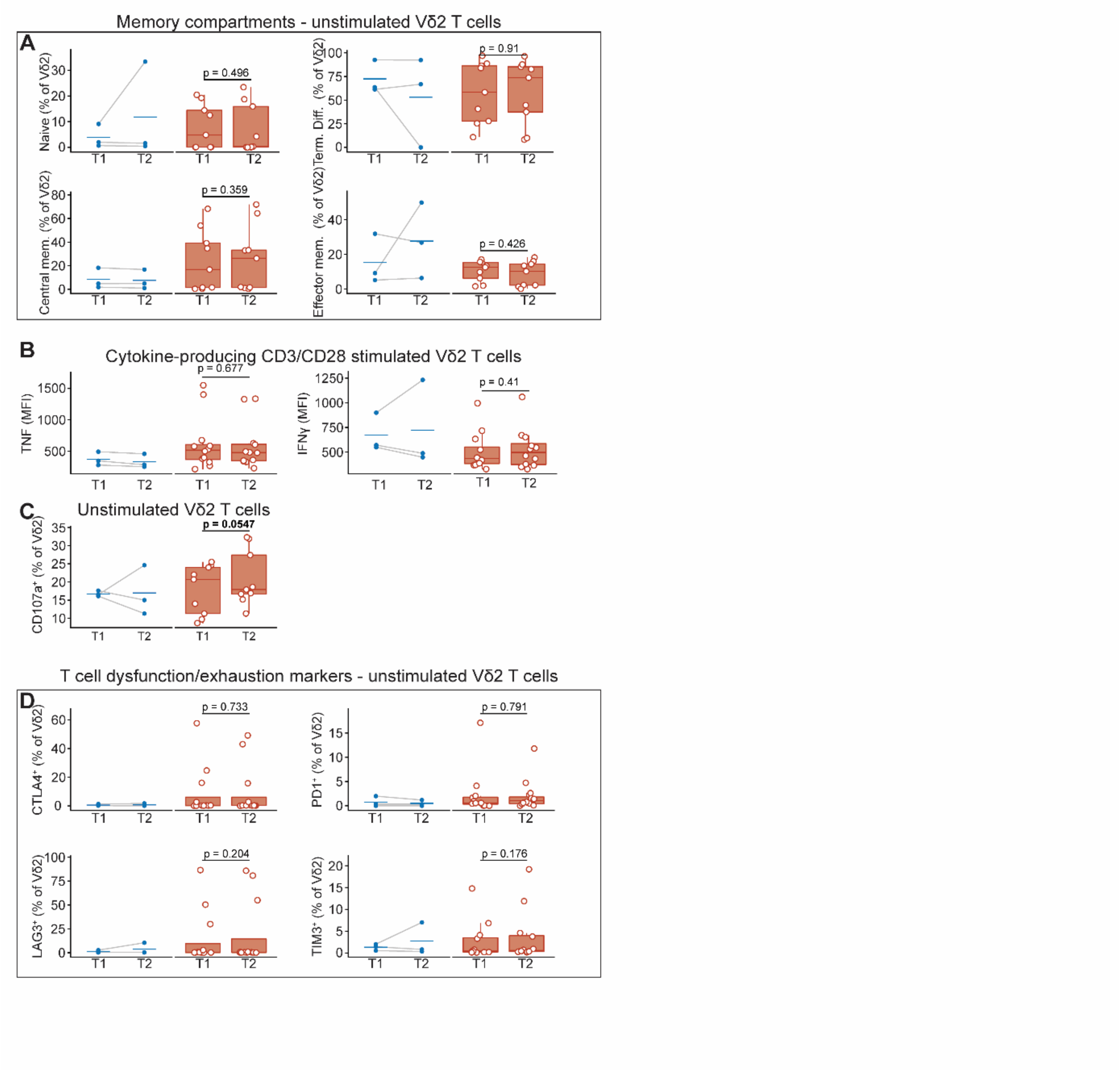

Figure S6: characterization of Vδ2 cells following MMR vaccination. (**A**) Proportions of memory compartments characterized by expression of CD27 and CD45RA. (**B**) Mean fluorescence intensities (MFI) of TNF and IFNγ in stimulated Vδ2 T cells. (**C**) Percentage of unstimulated Vδ2 T cells that stain positive for CD107a, a marker of cytotoxic degranulation. (**D**) Proportions of Vδ2 T cells that stain positive for markers commonly associated with T cell dysfunction (CTLA4, PD1, TIM3, LAG3).

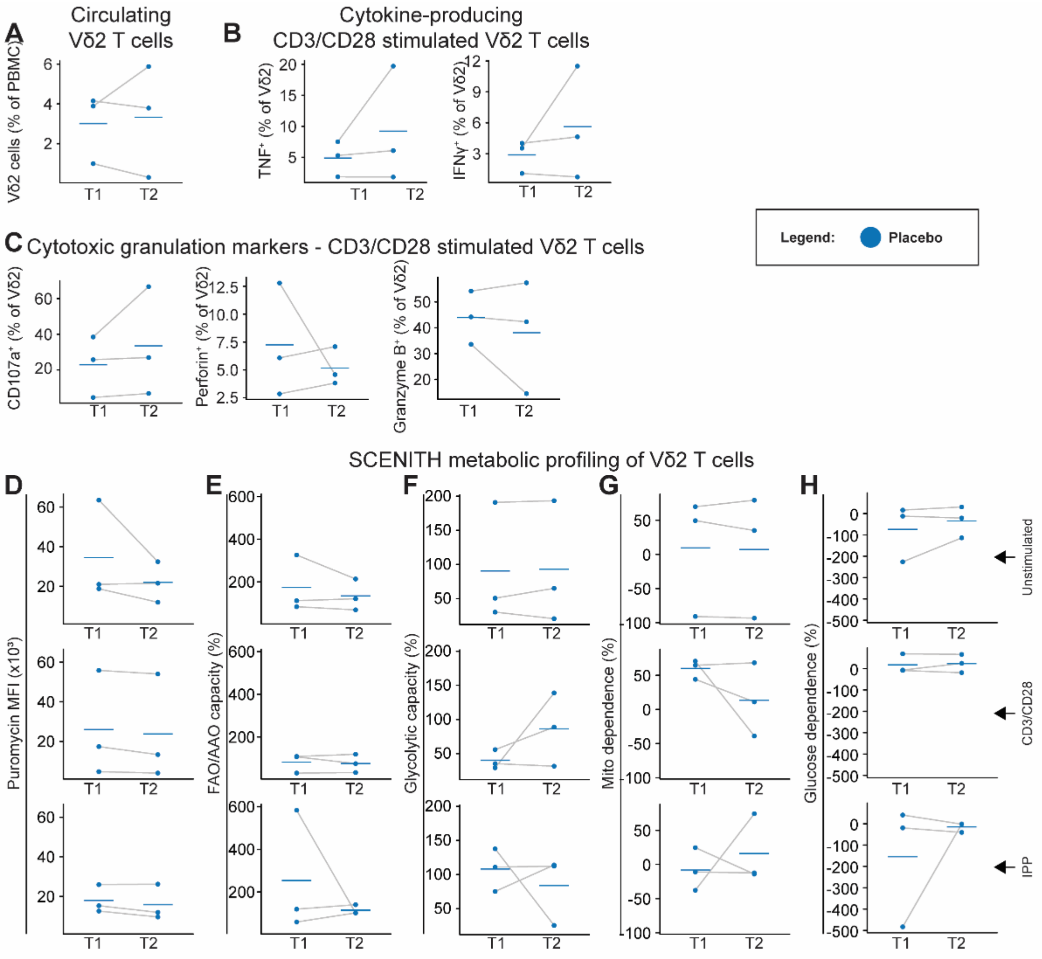

Figure S7. Functional and metabolic characterization of Vδ2 cells following placebo vaccination. (A) The percentage of Vδ2 T cells in isolated PBMCs. (B) The percentage of Vδ2 T cells that produce TNF or IFNγ following CD3/CD28 stimulation. (C) The percentage of Vδ2 T cells expressing markers of cytotoxic granule release (CD107a, left) or production (Granzyme B and perforin, middle and right). metabolic parameters by modified SCENITH^{TM} (https://www.scenith.com) calculated as in Argüello *et al.* (24): (D) puromycin incorporation, (E) FAO/AAO capacity, (F) Glycolytic capacity, (G) Mitochondrial dependence, (H) Glycolysis dependence. All parameters were measured by flow cytometry.

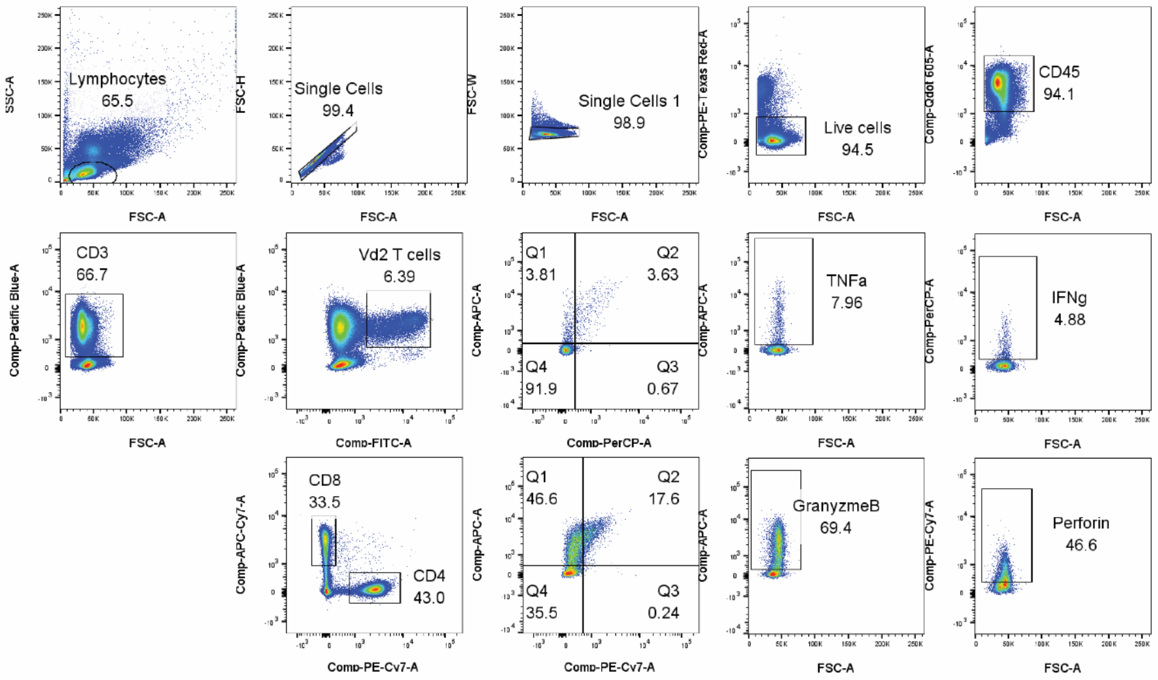

Figure S8. Visual representation of the gating strategy used for flow cytometry analyses of Vδ2 T cells. The gating strategy was as follows: events corresponding to lymphocyte size were selected based on FSC-A/SSC-A, followed by selection of single-cell events in subsequent FSC-H/FSC-A and FSC-W/FSC-A gates. Viable cells were selected by gating on viability-dye-negative cells. The subsequent analyses were all performed on $CD45^{+}CD3^{+}V\delta2^{+}$ cells.

Supplementary tables

Characteristic[*]	Placebo<br>n = 18	MMR<br>n = 21	p-value[†]
Sex, female	7 (38.89)	12 (57.14)	0.3406
Sex, male	11 (61.11)	9 (42.86)	
Age, years	25 ± 7.63	25 ± 7.38	0.876
BMI, kg/m^2	23.47 ± 1.89	23.45 ± 5.22	0.945

* Median +/- SD for continuous variables and n (%) for categorical variables

† Mann-Whitney U test for continuous variables and Fisher's exact test for categorical variables

Table S1: characteristics of the study population.

Stimulus	Concentration in experiment	Cat. No.	Manufacturer
E. coli LPS serotype O55:B5, further purified as in (*54*)	10 ng/ml	-	Prepared in-house by Heidi Lemmers, Raboudumc
Heat-killed *S. aureus* ATCC 25923	1 * 10^6 CFU/ml	-	Cultured and heat-killed in-house by Jelle Gerretsen, Radboudumc
Heat-killed *C. albicans* yeast, UC820 (ATCC MYA-3573), prepared as described in (*55*)	1 * 10^6 CFU/ml	-	Prepared in-house by Diletta Rosati, Radboudumc
Poly(I:C)	10 µg/ml	tlrl-pic	Invivogen
R848	3 µg/ml	tlrl-r848	Invivogen
Influenza A H1N1, prepared according to the methods in (*56*)	3.3 * 10^5 /ml	-	Kindly prepared by KLG, NR, PNO, LM, HS, OA, AB
SARS-CoV-2, prepared according to the methods in (*56*)	1.4 * 10^3 /ml	-	

Table S2: Stimuli used to assess PBMC cytokine production capacity.

Target	Fluorophore	Clone	Cat. No.	Manufacturer	Panel
Viability	Live-or-Dye Fixable Viability Stain	-	32006	Biotium	1
CD3	Pacific blue	UCHT1	300431	Biolegend	1
CD4	PE-Cy7	OKT4	317414	Biolegend	1
CD8	APC-Cy7	SK1	344746	Biolegend	1
CD45	BV605	HI30	304042	Biolegend	1
Vδ2 TCR	FITC	REA771	130-111-009	Miltenyi	1
Vδ1 TCR	PE	REA173	130-120-440	Miltenyi	1
TNF	APC	MAb11	502912	Biolegend	1
IFNγ	PerCP-Cy5.5	B27	560704	BD Pharmingen	1
Viability	Live/dead Fixable Aqua dead cell stain	-	L34957	Invitrogen	2
CD3	Pacific blue	UCHT1	300431	Biolegend	2
Vδ2 TCR	FITC	REA771	130-111-009	Miltenyi	2
Vδ1 TCR	PE	REA173	130-120-440	Miltenyi	2
CD4	BV605	OKT4	317438	Biolegend	2
CD8	APC-Cy7	SK1	344746	Biolegend	2
PD1	PerCP-Cy5.5	A17188 B	621613	BD Bioscience	2
Perforin	Pe-Cy7	dG9	308126	Biolegend	2
Granzyme B	AF647	GB11	515406	Biolegend	2
CD45	PE/Dazzle594	HI30	304052	Biolegend	2
Viability	Live/dead Fixable Aqua dead cell stain	-	L34957	Invitrogen	3
CD3	APC	UCHT1	300458	Biolegend	3
CD4	PE-Cy7	OKT4	317414	Biolegend	3
CD8	APC-Cy7	SK1	344746	Biolegend	3
Vδ1 TCR	Pacific blue	REA173	130-100-555	Miltenyi	3
Vδ2 TCR	FITC	REA771	130-111-009	Miltenyi	3
CD107a	PE	H4A3	328607	Biolegend	3
CTLA4	BV605	BNI3	369610	Biolegend	3
Lag3	PE/Dazzle594	11C3C6 5	369331	Biolegend	3
Viability	Live/dead Fixable Aqua dead cell stain	-	L34957	Invitrogen	4
CD45	PE/Dazzle594	HI30	304052	Biolegend	4
CD45RA	APC	HI100	304111	Biolegend	4
CD3	Pacific blue	UCHT1	300458	Biolegend	4
CD4	PE-Cy7	OKT4	317414	Biolegend	4
CD8	APC-Cy7	SK1	344746	Biolegend	4
Vδ1 TCR	PE	REA173	130-120-440	Miltenyi	4
Vδ2 TCR	FITC	REA771	130-111-009	Miltenyi	4
CD27	PerCP-Cy5.5	M-T271	560612	BD Bioscience	4
NKG2D	BV605	1D11	320832	Biolegend	4

Viability	Live-or-Dye Fixable Viability Stain	-	32006	Biotium	5
CD3	Pacific blue	UCHT1	300431	Biolegend	5
CD4	PE-Cy7	OKT4	317414	Biolegend	5
CD8	APC-Cy7	SK1	344746	Biolegend	5
CD45	BV605	HI30	304042	Biolegend	5
Vδ2 TCR	APC	REA771	130-111-009	Miltenyi	5
Vδ1 TCR	PE	REA173	130-120-440	Miltenyi	5
Puromycin	AF488	12D10	MABE343	Merck	5

Table S3: Antibodies used in the flow cytometric analyses of Vδ2 T cells.

54. M. Hirschfeld, Y. Ma, J. H. Weis, S. N. Vogel, J. J. Weis, Cutting Edge: Repurification of Lipopolysaccharide Eliminates Signaling Through Both Human and Murine Toll-Like Receptor 2. *The Journal of Immunology* **165**, 618 (2000).
55. D. Rosati *et al.*, Activation of cytokine responses by Candida africana. *Med Mycol* **60**, (2022).
56. P. A. Debisarun *et al.*, Induction of trained immunity by influenza vaccination - impact on COVID-19. *PLOS Pathogens* **17**, e1009928 (2021).
